# Copine A plays a role in the differentiation of stalk cells and the initiation of culmination in *Dictyostelium *development

**DOI:** 10.1186/1471-213X-10-59

**Published:** 2010-06-02

**Authors:** Tasha S Smith, Jaimie M Pineda, Alex C Donaghy, Cynthia K Damer

**Affiliations:** 1 Department of Biology, Central Michigan University, Mount Pleasant, Michigan 48859 USA

## Abstract

**Background:**

Copines are calcium-dependent phospholipid-binding proteins found in diverse eukaryotic organisms. We are studying the function of copines in *Dictyostelium discoideum*, a single-celled amoeba that undergoes cell differentiation and morphogenesis to form multicellular fruiting bodies when placed in starvation conditions. Previously, we showed that *Dictyostelium *cells lacking the copine A (*cpnA*) gene are not able to complete the developmental cycle, arresting at the slug stage. The aim of this study is to further characterize the developmental defect of the *cpnA*- cells.

**Results:**

Time-lapse imaging revealed that *cpnA*- cells exhibited delayed aggregation and made large mounds that formed one large slug as compared to the smaller slugs of the wild-type cells. While the prespore cell patterning appeared to be normal within the *cpnA*- slugs, the prestalk cell patterning was different from wild-type. When *cpnA*- cells were mixed with a small percentage of wild-type cells, chimeric fruiting bodies with short stalks formed. When a small percentage of *cpnA*- cells was mixed with wild-type cells, the *cpnA*- cells labeled with GFP were found located throughout the chimeric slug and in both the stalk and sporehead of the fruiting bodies. However, there appeared to be a small bias towards *cpnA*- cells becoming spore cells. When *cpnA*- cells were developed in buffer containing EGTA, they were also able to differentiate into either stalk or spore cells to form fruiting bodies with short stalks.

**Conclusions:**

Our results indicate that CpnA is involved in the regulation of aggregation, slug size, and culmination during *Dictyostelium *development. More specifically, CpnA appears to be involved in the function and differentiation of prestalk cells and plays a role in a calcium-regulated signaling pathway critical to triggering the initiation of culmination.

## Background

Copines are highly conserved calcium-dependent membrane binding proteins found in many eukaryotic organisms including *Paramecium*, *Dictyostelium*, *Arabidopsis*, *C. elegans*, mice, and humans [1-5]. The copine family is characterized as having two C2 domains in the N-terminal half of the protein followed by an A domain in the C-terminal half. Following the A domain, copines have a variable length C-terminal domain, which may confer unique characteristics to the different copine family members [1].

The C2 domain is a calcium-dependent phospholipid-binding motif originally identified in protein kinase C. Single and multiple copies of C2 domains are found in a large number of eukaryotic proteins. Most proteins containing a single C2 domain are involved in signaling pathways; examples include protein kinases, lipid kinases, phospholipases, and GTPase activating proteins. In contrast, most proteins that have multiple C2 domains are involved in membrane trafficking and exocytosis. Some examples of multiple C2 domain proteins are synaptotagmin, rabphilin, DOC2, each of which has two C2 domains, and munc13, which has three C2 domains [6].

The A domain is similar in sequence to the von Willebrand A (VWA) domain found in integrins. The VWA domain is named from the von Willebrand Factor, a plasma and extracellular matrix protein. VWA domains have been studied in integrins and several extracellular matrix proteins and appear to function as protein-binding domains [3]. Copines were the first intracellular proteins to be identified as having a VWA domain [1]. However, a more recent sequence database search for VWA domains revealed that VWA domains are found in several other intracellular proteins present in eukaryotes [3].

The combination of a domain typically found in extracellular domains/proteins and a domain typically found in intracellular proteins involved in calcium-mediated functions makes these proteins unique and interesting to study. In addition, the wide array of organisms ranging from single-celled organisms to humans in which copines are found suggest that copines carry out fundamental functions important in most eukaryotic cells. Although the exact function of copines is not known, a growing body of evidence suggests that copines may mediate an array of cellular processes by conferring calcium regulation to various signaling pathways [3,7-9].

A general hypothesis proposed by Tomsig et al. [10] for how copines may regulate signaling pathways is that specific copines interact with other cellular proteins through their A domains and then either deliver soluble target proteins to membranes or regulate the function of a membrane protein through the action of the C2 domains in response to a rise in intracellular calcium. Tomsig et al. [10] identified more than 20 distinct potential targets of A domains of human copines I, II, and IV using a yeast two-hybrid screen. Among the proteins that were found to associate with human copine A domains were various regulators of phosphorylation, transcription, ubiquitination, cytoskeleton, exocytosis, and mitosis, suggesting that copines carry out many diverse functions.

Ramsey et al. [11] showed that copine I inhibits NF-κB regulated transcription in response to TNFα in prostate cancer cell lines by inducing the proteolysis of the N-terminus of p65, one of the subunits of NF-κB. These authors suggest the copine I may associate through its A domain with protease-containing protein complexes to regulate proteolysis of NF-κB subunits. Therefore, copines may not only directly interact with and regulate the activity of cytosolic proteins, but also regulate the activity of many proteins at the level of transcription.

Copine mutants in *Arabidopsis *have defects in growth and development and exhibit increased cell death and disease resistance [9,12-15]. The BON1/CPN1 copine protein in *Arabidopsis *negatively regulates the activity of the disease resistance R gene SNC1 at the transcriptional level [16,17]. In addition, up-regulation of BON1/CPN1 expression can be induced by bacterial pathogen inoculation and calcium influx produced by treatment with a calcium ionophore [7]. Therefore, BON1/CPN1 may function to suppress the activation of plant defense signaling in response to increases in intracellular calcium, thereby inhibiting cell death in response to pathogen detection.

Copines in *C. elegans *have not been studied directly. However, mutations in *gem-4*, a copine gene, were found to suppress loss-of-function alleles of the *gon-2 *gene. GON-2 is a cation channel required for division of the four postembryonic gonad-precursor cells in *C. elegans *[18]. GON-2 is a member of the family of TRPM (transient receptor potential melastatin) ion channels. GON-2 in the intestinal epithelial appears to be required for an outwardly rectifying calcium current and functions in IP_3 _signaling to regulate intestinal calcium oscillations [19]. GEM-4 tagged with GFP was found localized to the plasma membrane and may negatively regulate the activity of GON-2 directly. In another study, a different copine protein was found to associate with the synaptic nicotinic acetylcholine receptor and the inactivation of the copine gene by RNAi resulted in nicotine resistance. The nicotine resistance appeared to be due to a reduction in synaptic nicotinic receptor expression [20]. These two studies indicate a role for copines in regulating the activity and/or expression of two different types of plasma membrane calcium channels in *C. elegans*.

We are studying the function of copine proteins in the simple eukaryote *Dictyostelium discoideum*. *Dictyostelium *can live independently as single-celled amoebae, yet can also undergo cell differentiation and morphogenesis to form multicellular fruiting bodies when placed in starvation conditions. Upon starvation, cells undergo chemotaxis in response to periodic waves of secreted cAMP and aggregate into hemispherical mounds. Next, a tip is formed on the mound that elongates to give rise to a finger-shaped structure. This finger may topple over and migrate as a slug before developing into a fruiting body consisting of a dead stalk with a spore mass on top. During the final stage of development, called culmination, a dramatic change in morphology occurs and cells terminally differentiate into spore or stalk cells. Associated with differentiation and morphological changes during development is a regulated program of gene expression brought on by a variety of secreted factors [21].

We have identified six copine genes in the *Dictyostelium *genome and have focused our study on one of these genes, *cpnA *[5]. Our previous studies showed that CpnA is required for normal development and the developmental expression patterns of several of the other copine genes suggest they too may be regulators of development. Cells lacking the *cpnA *gene are arrested during development in the slug stage and do not culminate into fruiting bodies [22]. In this current study, we have characterized the developmental phenotype of *cpnA*- cells further. Time-lapse imaging of developing *cpnA*- cells indicated that *cpnA *has roles in regulating aggregation, slug size, and culmination. Mixing wild-type cells with *cpnA*- cells allowed *cpnA*- cells to develop into fruiting bodies forming differentiated stalk or spores cells, indicating that *cpnA*- cells are able to respond to signals from wild-type cells. In addition, developing *cpnA*- cells in a reduced calcium environment allowed *cpnA*- cells to develop into fruiting bodies suggesting that CpnA may have a role in mediating calcium-regulated signaling pathways important in development.

## Results

### *cpnA*- cells are arrested in the slug stage of development

**Figure 1:**
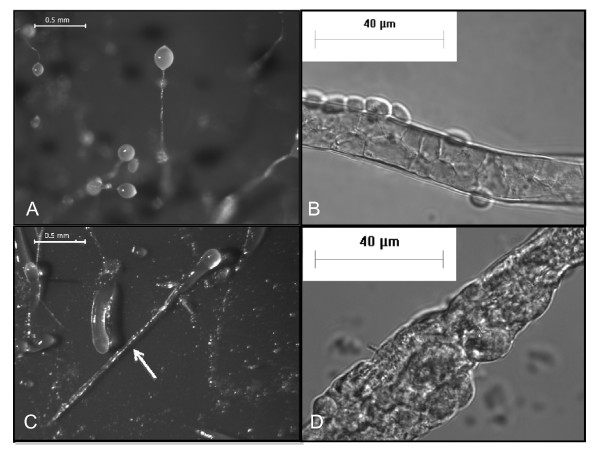
***cpnA*- cells are arrested in the slug stage of development**. Wild-type (A) and *cpnA*- (C) *Dictyostelium *cells were plated on black filters at 5 × 10^7 ^cells/mL in starvation buffer and developed for 48 hours. Images were taken using a Leica dissecting microscope at 40× magnification (scale bar = 0.5 mm). Wild-type (B) and *cpnA*- (D) stalk structures were removed from the filters, placed in glass bottom dishes, and imaged using a Nikon fluorescence microscope at 1000× (B) and 600× (D) magnification with DIC optics (scale bar = 40 μm).

In a previous study, we showed that *Dictyostelium *cells lacking the *cpnA *gene exhibit developmental defects [22]. *cpnA*- cells become arrested in the slug stage of development, indicating that *cpnA *is necessary for normal development, specifically in the last stage of development called culmination. We looked at the development of three independent *cpnA*- clones on filters and they each exhibited this same developmental defect [22]. In this study, we further investigated the role of *cpnA *in development. Wild-type and *cpnA*- cells were plated on black filters and allowed to develop for 24-48 hours; *Dictyostelium *cells placed in starvation buffer on filters at a particular density will complete their development in a characteristic 24-hour period. Wild-type cells formed fruiting bodies as shown in Figure 1A, while *cpnA*- cells were either arrested in the finger/slug stage or made structures with what appeared to be a slug or finger-like structure on top of a stalk-like structure (Figure 1C, arrow). However, when these stalk-like structures were observed at a higher magnification, it was clear that they were not made up of differentiated stalk cells (Figure 1D). Wild-type stalk cells undergo programmed cell death and become vacuolated as shown in Figure 1B.

### Time-lapse imaging revealed that *cpnA*- cells form larger mounds that develop into fewer, larger slugs

**Figure 2:**
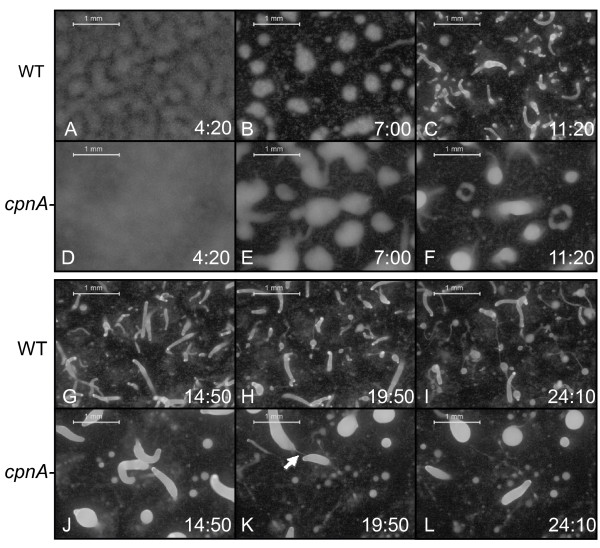
**Time-lapse imaging revealed that *cpnA*- cells are delayed in aggregation and form larger mounds that develop into fewer, larger slugs**. Wild-type (A-C, G-I) and *cpnA*- (D-F, J-L) *Dictyostelium *cells expressing GFP were plated on black filters in starvation buffer at 5 × 10^7 ^cells/mL and imaged every 10 minutes for 28 hours using a Leica dissecting fluorescence microscope at 20× magnification (scale bar = 1 mm). Hours:minutes in development are given in the lower right corner of each image. Arrow (K) points to stalk-like structure. Also see time-lapse movies in Additional Files 1 and 2.

To further characterize the developmental defects of *cpnA*- cells, we used fluorescence time-lapse microscopy to image both wild-type and *cpnA*- cells during development. Cells expressing green fluorescent protein (GFP) under the *act15 *promoter were plated on filters and imaged at ten-minute intervals for 28 hours (Figure 2 and Additional Files 1 and 2). Time-lapse imaging revealed that the *cpnA*- cells aggregated into mounds with a slight delay as compared to the wild-type parental strain (Figure 2A and 2D). The mounds of *cpnA*- cells were larger than the wild-type mounds (Figure 2B and 2E) and did not break up into several fingers, as did the wild-type parental strain mounds (Figure 2C and 2F). Instead, each mound of *cpnA*- cells formed one large slug (Figure 2G-L). At approximately 20 hours of development, wild-type structures began culmination (Figure 2H). Interestingly, some of the large slugs of the *cpnA*- cells also appeared to begin culmination, in that a stalk-like structure was formed (Figure 2K, arrow). However, a sorus or sporehead did not form and the slug seemed to be able to move away from the stalk-like structure built underneath it (Figure 2K). At 24 hours, there were many fruiting bodies on the wild-type plate and none on the *cpnA*- cell plate (Figure 2I and 2L).

### Prespore and prestalk cell patterning in *cpnA*- cells

During *Dictyostelium *development, cells differentiate into two main cell types, stalk cells and spores, due to changes in gene expression that begin early in development. Approximately 20% of the cells within a slug become stalk cells, while 80% become spores [21]. Cells that will become stalk cells, called prestalk cells, and those that will become spores, called prespore cells, can be distinguished by particular prespore and prestalk marker genes [23]. We used three of these marker genes to determine if the pattern and localization of prestalk and prespore cells are disrupted in the *cpnA*- cells. Wild-type and *cpnA*- cells were transformed with plasmids that contained the promoter sequence for *ecmAO*, a prestalk marker gene, *ecmB*, another prestalk marker gene, or *pspA*, a prespore marker gene, upstream of the *lacZ *gene [24-26]. The transformed cells were developed on filters and then fixed and stained with X-Gal to reveal either prespore or prestalk patterning at various stages of development.

**Figure 3:**
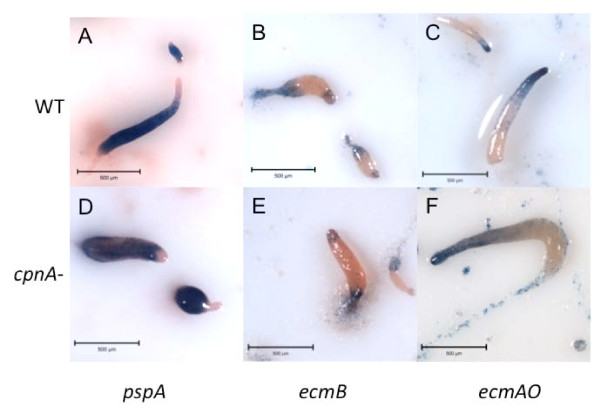
**Prestalk and prespore cell patterning in *cpnA*- slugs**. Wild-type (A-C) and *cpnA*- (D-F) *Dictyostelium *cells transformed with the *pspA*-Gal (A, D) *ecmAO*-Gal (B, E), and *ecmB*-Gal (C, F) plasmids were plated on white filters in starvation buffer at 5 × 10^7 ^cells/mL and allowed to develop for 24 hours. At ~16 hours of development, slug structures were fixed and stained with X-Gal to reveal prestalk and prespore patterning. Images were taken with the Nikon SMZ1500 dissecting microscope equipped with a SPOT Flex colored camera using the SPOT digital imaging software (scale bar = 500 μm).

Prespore patterning of the *pspA *promoter appeared to be normal in the *cpnA*- slugs, with the *pspA *expressing cells found in the posterior four-fifths of the slug as seen in the parental wild-type cells (Figure 3A and 3D). The prestalk specific *ecmB *promoter, which is active in specific types of prestalk cells including pstB and pstAB cells, was expressed in a region within the anterior one-fifth of the slug, cells scattered throughout the slug, and cells in the rear of the slug, in both the slugs of the parental wild-type strain and the *cpnA*- strain (Figure 3B and 3E). However, staining in the anterior of the slug was seen less often in the *cpnA*- cells, which may be because the *cpnA*- cells spend more time as slugs; pstAB cells will move to the rear and be shed from the slug during long periods of migration [27]. The prestalk specific promoter, *ecmAO*, is active in both pstA and pstO cells found in the anterior portion of the slug and was observed in this region of the wild-type slug (Figure 3C). In the *cpnA*- slugs, the *ecmAO *promoter was active in the anterior region of the slug like the wild-type slugs, but also appeared to be active in the rear of the *cpnA*- slug and cells within the slug trail (Figure 3F). The X-Gal staining of cells at the posterior end of the slug and the slug trail was not observed in the wild-type slug (Figure 3C).

### Wild-type cells can rescue the culmination defect of *cpnA*- cells

**Figure 4:**
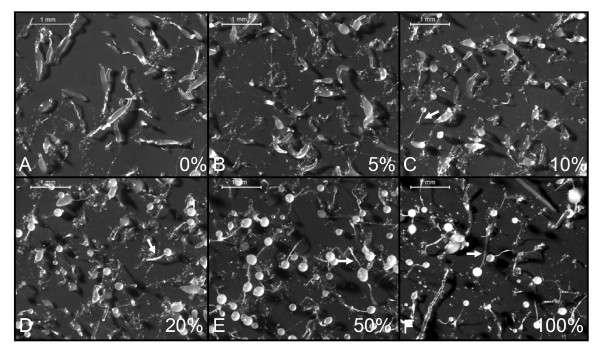
**Mixing with 10% wild-type cells begins to rescue the culmination defect of *cpnA*- cells**. Wild-type and *cpnA- Dictyostelium *cells were mixed at various ratios, plated on black filters in starvation buffer at 5 × 10^7 ^cells/mL, and allowed to develop for 24 hours. The wild-type cell percentage is given in the lower right of each image. Images were taken using a Leica dissecting microscope at 20× magnification (scale bar = 1 mm). Arrows (C-F) point to stalks.

To determine whether mixing wild-type cells with *cpnA*- cells rescues the developmental defect of *cpnA*- cells, we mixed wild-type cells and *cpnA*- cells at various ratios and plated the cell mixtures in starvation buffer on filters (Figure 4). We found that mixing 10% wild-type cells with *cpnA*- cells resulted in a few small fruiting bodies with abnormally short stalks on each plate (Figure 4C, see arrow). Mixing *cpnA*- cells with 20% wild-type cells resulted in fruiting bodies all over the plates; however, again the fruiting bodies had shorter than normal stalks (Figure 4D). As the ratio of wild-type cells was increased, the stalks also lengthened and at 50% wild-type cells, the fruiting bodies looked similar to those formed from 100% wild-type cells (Figure 4E and 4F). In addition, if cells were allowed to develop for 48 hours instead of 24 hours as shown, small fruiting bodies were observed on the 5% wild-type plate and found all over the 10% wild-type plate (data not shown).

**Figure 5:**
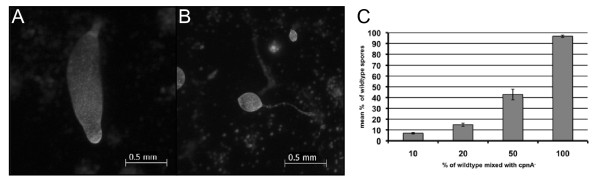
**Wild-type cells differentiate into stalk or spore cells when mixed with *cpnA*- cells**. Wild-type cells expressing GFP (10%) were mixed with *cpnA*- cells (90%), plated on black filters in starvation buffer at 5 × 10^7 ^cells/mL, and allowed to develop for 24 hours. Fluorescent images of chimeric slugs (A) and fruiting bodies (B) were taken with a Leica dissecting microscope at 50× magnification (scale bar = 0.5 mm). (C) Wild-type cells expressing GFP and *cpnA*- cells were mixed at various ratios and allowed to develop. Fruiting bodies were removed from the filter, placed on glass bottom dishes, and imaged with a Nikon fluorescence microscope. The percentage of fluorescent spores from each ratio was calculated five times and averaged. Error bars indicate standard error.

These data indicate that mixing a small percentage of wild-type cells with *cpnA*- cells allows the *cpnA*- cells to differentiate into either stalk or spore cells to form fruiting bodies. However, if the *cpnA*- cells are specifically defective in either stalk or spore cell differentiation, then it is possible that wild-type cells were able to rescue the culmination defect by preferentially becoming either the stalk cells or the spores formed in these small chimeric fruiting bodies. Therefore, we did the same mixing experiment, but with wild-type cells labeled with GFP. Fluorescence microscopy images of chimeric slugs and fruiting bodies revealed that wild-type cells were not found preferentially in the prestalk or prespore areas of the slug or in the stalk or sporehead of the fruiting body; instead wild-type cells were found throughout the whole slug and in both the stalk and sporehead of the fruiting bodies. A slug and fruiting body formed by the mixture of 10% wild-type cells labeled with GFP and 90% unlabeled *cpnA*- cells are shown in Figure 5A and 5B. To determine the exact make-up of the spores in the chimeric fruiting bodies, we removed the spore heads, placed them on glass-bottom dishes, and determined the percentage of wild-type spores, which were labeled with GFP. We found the percentage of wild-type spores within the sporehead was slightly lower on average, but similar to the percentage of wild-type cells originally mixed with *cpnA*- cells (Figure 5C). This finding indicates that both wild-type and *cpnA*- cells were almost equally likely to become spores and that the presence of a small percentage of wild-type cells allowed *cpnA*- cells to differentiate into spores. However, the shorter stalks of the chimeric fruiting bodies formed from the lower wild-type ratio cell mixtures suggest that *cpnA*- cells have a defect in stalk cell differentiation.

### *cpnA*- cells mixed with wild-type cells can become either stalk or spore cells

**Figure 6:**
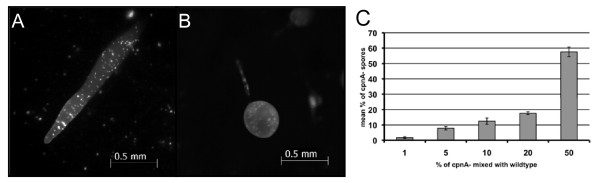
***cpnA*- cell are able to differentiate into either stalk or spore cells when mixed with wild-type cells**. *cpnA*- cells expressing GFP (10%) were mixed with wild-type cells (90%), plated on black filters in starvation buffer at 5 × 10^7 ^cells/mL, and allowed to develop for 24 hours. Fluorescent images of chimeric slugs (A) and fruiting bodies (B) were taken with a Leica dissecting microscope at 50× magnification (scale bar = 0.5 mm). (C) *cpnA*- cells expressing GFP and wild-type cells were mixed at various ratios and allowed to develop. Fruiting bodies were removed from the filter, placed on glass bottom dishes, and imaged with a Nikon fluorescence microscope. The percentage of fluorescent spores from each ratio was calculated five times and averaged. Error bars indicate standard error.

We also performed the reverse experiment in which a small percentage of *cpnA*- cells expressing GFP was mixed with wild-type cells. Fluorescence microscopy revealed that *cpnA*- cells expressing GFP were found throughout the slug (Figure 6A) and in both the sporehead and the stalk of fruiting bodies (Figure 6B) indicating that *cpnA*- cells have the ability to differentiate into either spores or stalk cells when mixed with wild-type cells. However, we found that at all percentages except for twenty, the percentage of *cpnA*- cells within the sporehead was slightly higher than the percentage of *cpnA*- cells in the original mixture of cells (Figure 6C).

One notable difference between the two types of mixing experiments was that the *cpnA*- cells expressing GFP appeared to form aggregates within the mostly wild-type slug (Figure 6A). Therefore, we did two additional types of mixing experiments: 10% wild-type/GFP cells mixed with 90% unlabeled wild-type cells and 10% *cpnA*-/GFP cells mixed with 90% unlabeled *cpnA*- cells. Both of these types of chimeric slugs resulted in diffuse fluorescence throughout the slug similar to the slug shown in Figure 5A (data not shown). These data suggest that *cpnA*- cells may be more adhesive than the wild-type cells and thus, adhere to each other more strongly and move within the slug together. If *cpnA*- cells are more adhesive than wild-type cells, then they may not break up into smaller streams or mounds during aggregation, which would explain the large slug phenotype.

**Figure 7:**
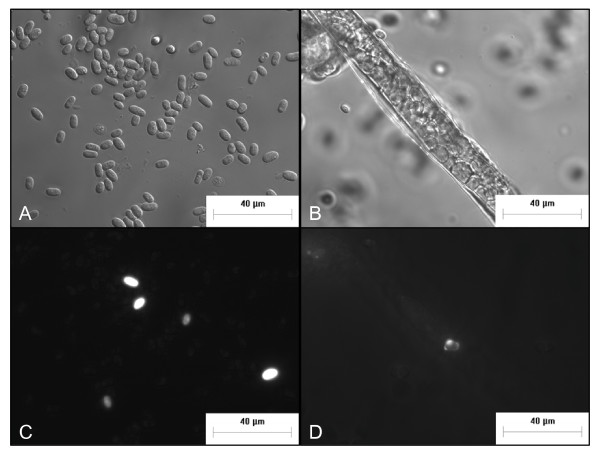
***cpnA*- cells can differentiate into morphologically normal stalk and spore cells**. *cpnA*- cells expressing GFP (5%) and wild-type cells (95%) were mixed, plated on black filters in starvation buffer at 5 × 10^7 ^cells/mL, and allowed to develop for 24 hours. Fruiting bodies were removed from the filter and placed in glass bottom dishes. Spores (A, C) and stalks (B, D) were imaged with DIC (A, B) and fluorescence (C, D) microscopy using a Nikon fluorescence microscope at 600× magnification (scale bar = 40 μm).

We also took fruiting bodies from the plates that contained 5% *cpnA*-/GFP cells mixed with 95% wild-type unlabeled cells, and placed them on slides to image them at a higher magnification. Using differential interference contrast (DIC) and fluorescence imaging, we found that the *cpnA*- cells appeared to form morphologically normal spore and stalk cells (Figure 7). The results of both types of mixing experiments indicate that *cpnA*- cells are able to differentiate into either spore or stalk cells in the presence of wild-type cells.

### *cpnA*- cells in reduced calcium environment are able to make fruiting bodies with short stalks

**Figure 8:**
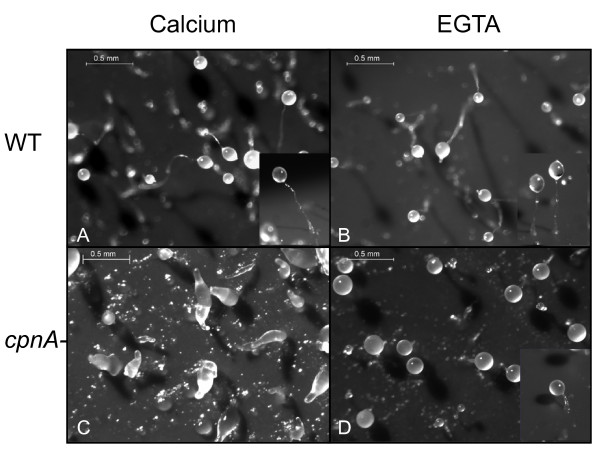
**In reduced calcium conditions, *cpnA*- cells form fruiting bodies with short stalks**. Wild-type (A, B) and *cpnA*- (C, D) cells were plated on black filters in starvation buffer with either 2 mM calcium (A, C) or 2 mM EGTA (B, D) at 5 × 10^7 ^cells/mL, and allowed to develop for 48 hours. Images were taken using a Leica dissecting microscope at 40× magnification (scale bar = 0.5 mm). Insets are side views of fruiting bodies to show stalk length at the same magnification.

**Figure 9:**
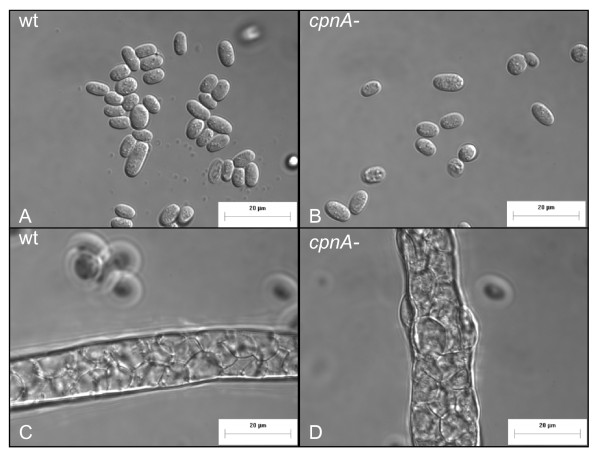
***cpnA*- cells can differentiate into morphologically normal spore and stalk cells in reduced calcium conditions**. Wild-type (A, C) and *cpnA*- (B, D) cells were allowed to develop in starvation buffer containing EGTA for 48 hours. Fruiting bodies were removed from filters, placed on glass bottom dishes, and imaged with a Nikon microscope at 1000× magnification (scale bar = 20 μm).

Because CpnA has two C2 domains and was previously shown to bind to membranes in the presence of calcium [5], we examined whether changes in available calcium affected the development of *cpnA*- cells. Wild-type and *cpnA*- cells were developed in starvation buffers containing various amounts of calcium or the calcium chelator, EGTA. We found that increasing the amount of calcium in the buffer up to 5 mM or decreasing the amount of calcium by not adding any calcium to the buffer did not greatly affect the development of wild-type or *cpnA*- cells. However, when *cpnA*- cells were developed in buffer containing 2 mM EGTA to chelate calcium ions, they were able to form fruiting bodies, albeit with abnormally short stalks (Figure 8D). To determine whether this EGTA result was due to the chelation of calcium ions, we also developed *cpnA*- cells in buffer containing 2 mM EGTA and 4 mM CaCl_2_. Adding back calcium in the presence of EGTA did not result in fruiting bodies; instead, *cpnA*- cells arrested in the slug/finger stage (data not shown). The short-stalked fruiting bodies made up of *cpnA*- cells consisted of morphologically normal stalk and spores cells (Figure 9). In addition, spores formed by the *cpnA*- cells developed in buffer containing EGTA were able to germinate when placed in nutrient media (data not shown). These data show that lowering the amount of calcium available in the environment allowed the *cpnA*- cells to differentiate into either stalk or spore cells. Shorter stalks were observed for both the wild-type and *cpnA*- fruiting bodies when developed in EGTA, although the *cpnA*- cells formed much shorter stalks than the wild-type.

## Discussion

In a previous study we showed that *Dictyostelium *cells lacking the *cpnA *gene were arrested at the slug stage of development. In the current study, we further characterized the developmental phenotype of *cpnA*- cells. Time-lapse imaging revealed that *cpnA*- cells exhibited delayed aggregation and formed large mounds that became larger than normal slugs. The *cpnA*- slugs were able to migrate, but did not culminate into fruiting bodies. These observations indicate that CpnA plays a role in regulating aggregation and slug size and is necessary for culmination. In addition to these phenotypic observations of *cpnA*- cells, we carried out several experiments to further explore the role of *cpnA *in cell differentiation and culmination.

Using prespore and prestalk marker genes, we found that *cpnA*- cells were able to achieve what appeared to be normal patterning of prespore cells within the developing slug prior to the culmination stage. In addition, there did not appear to be a major disruption in prestalk cell patterning in the *cpnA*- slugs prior to culmination; however, the prestalk patterning in the *cpnA*- slugs was not completely normal. The *ecmB *marker, which labels pstB and pstAB cells found in different areas of the anterior portion of the slug, was observed less often in the *cpnA*- cells as compared with wild-type cells. The *ecmAO *marker was observed at the anterior and posterior ends of the slug in *cpnA*- cells, while only at the anterior end in wild-type cells, suggesting that the EcmA expressing cells moved to the back of the slug and were sloughed off instead of differentiating into stalk cells. The cells that make up the anterior tip of the slug in the prestalk area are thought to be responsible for sensing whether the environment is suitable for culmination [28]. In normal development, if conditions are favorable for culmination, pstAB cells, which express both EcmA and EcmB, initiate culmination by producing the stalk tube. The surrounding pstA cells are then recruited into the tube as it elongates downward to the substratum. The maturing stalk cells within the tube secrete factors that signal to the prespore cells to begin differentiation into mature spore cells, and the spore cells begin their movement up the stalk. In the migratory slug in which culmination is inhibited, the pstAB cells will periodically move to the rear and be shed from the slug [23,27]. It is likely that the cause for the observed differences between *cpnA*- slugs and wild-type slugs in prestalk cell patterning is that *cpnA*- slugs cannot culminate. The inability of *cpnA*- slugs to culminate suggests that CpnA may function in the prestalk cells at the tip of slug as a component of the signaling pathway that triggers the initiation of culmination.

We carried out mixing experiments to test the hypothesis that CpnA is involved in regulating cell-to-cell signaling during *Dictyostelium *development. Because mixing wild-type cells with *cpnA*- cells was able to rescue the culmination defect in *cpnA*- cells, we were able to further explore the role of CpnA in cell differentiation. If CpnA is involved in the regulation of a signaling molecule that is either secreted or found on the plasma membrane, then a small percentage of wild-type cells may be able to rescue the culmination defect observed in the *cpnA*- cells. We found that mixing *cpnA*- cells with as little as 10% wild-type cells could partially rescue the culmination defect, in that these mixed cell populations formed fruiting bodies with viable spores, but with shorter than normal stalks. The wild-type cells mixed with *cpnA*- cells did not appear to preferentially become spores or stalk cells indicating that the presence of wild-type cells allowed *cpnA*- cells to differentiate into either spores or stalk cells.

To test this idea more directly, we also performed the reverse mixing experiment and found that when a small percentage of *cpnA*- cells was mixed with wild-type cells, *cpnA*- cells were able to become spore or stalk cells with a small bias towards becoming spore cells. Thus, the wild-type cells in the mixed/chimeric population slug may provide sufficient quantities of a secreted molecule to allow the whole slug to progress to culmination. Conversely, the wild-type cells may be able to metabolize or uptake a signaling molecule that inhibits culmination. Alternatively, the wildtype cells may provide enough normal functioning prestalk cells in the tip of the slug to trigger the slug to begin culmination. Once culmination begins, *cpnA*- cells are then able to undergo cell differentiation to form spore or stalk cells. However, results from each type of mixing experiment suggest that *cpnA*- cells may have a specific defect in stalk cell differentiation: the short stalks of the chimeric fruiting bodies formed from mixtures of mostly *cpnA*- cells and the small bias of *cpnA*- cells towards becoming spore cells in mixtures of mostly wild-type cells.

Because the CpnA protein contains C2 domains, we hypothesized that CpnA may function in calcium-regulated signaling either by controlling transient changes in calcium concentrations or by responding to changes in calcium concentrations. To search for possible defects in calcium signaling in *cpnA*- cells, we developed *cpnA*- cells and wild-type cells in buffers containing various concentrations of calcium. In buffer containing no additional calcium and 2 mM EGTA to chelate calcium ions, both the wild-type cells and *cpnA*- cells developed into fruiting bodies. The wild-type cells formed fruiting bodies with slightly shorter stalks than when developed in the absence of EGTA, while the *cpnA*- cells formed fruiting bodies with very short stalks. It is not unexpected that wild-type cells formed shorter stalks in buffer with EGTA given that other studies with *Dictyostelium *cells have shown that a larger percentage of cells become prespore cells when developed in EGTA [29]. Presumably this is because stalk cell differentiation requires higher levels of calcium [30-32]; prestalk cells also exhibit calcium wave oscillations not observed in prespore cells [33]. Gross [34] argues that calcium released from intracellular acidic stores is suppressed in prespore cells and active in prestalk cells leading to differences in gene expression in these two cell types necessary for differentiation. In a previous study, we speculated that CpnA may be specifically involved in stalk cell differentiation because a GFP-tagged version of CpnA was shown to transiently bind to membranes in an oscillatory way in a small subset of starved cells [5]. Although *cpnA*- cells were able to become stalk cells in the presence of wild-type cells and EGTA, in both cases, the stalks within the fruiting bodies were short. These results indicate that fewer *cpnA*- cells differentiated into stalk cells than normal and points to a specific defect in stalk cell function and differentiation.

Studies using *Arabidopsis *suggest that the copine BON1/CPN1 functions to suppress the activation of plant defense signaling in response to increases in intracellular calcium [7]. The role of CpnA in *Dictyostelium *development may be similar to what has been observed in *Arabidopsis*, in that it negatively regulates responses to increases in intracellular calcium. Therefore, the absence of CpnA may result in a hyper-response to calcium, leading to the observed defect in development. Adding EGTA to the developmental buffer will not only reduce extracellular calcium concentrations, but will also reduce intracellular calcium stores in *Dictyostelium *[35]. Therefore, if CpnA functions to negatively regulate its target protein in response to increases in intracellular calcium, then adding EGTA to the development buffer may allow for the almost normal activity of CpnA target proteins in the absence of CpnA. Alternatively, CpnA may be directly involved in controlling calcium levels and participate in the transmission of an extracellular signal by regulating the activity, trafficking, or transcription of a calcium channel or calcium channel-linked receptor as copines have been found to do in *C. elegans *[18,20]. Adding EGTA to the developmental buffer may bring intracellular calcium levels to a more normal level in the absence of CpnA.

## Conclusions

The calcium-dependent membrane-binding protein CpnA plays a role in regulating aggregation, slug size, and culmination during *Dictyostelium *development. Although CpnA does not appear to be essential for prespore/prestalk patterning in the early stages of development prior to culmination, our results indicate that CpnA plays a role in calcium-regulated signaling pathways necessary for stalk cell differentiation and the initiation of culmination.

## Methods

### Dictyostelium Strains

The *cpnA*- null mutant cells were made as previously described [22] by homologous recombination using *Dictyostelium *NC4A2 cells, an axenic strain derived from the wild-type NC4 strain, as the parental strain [36]. The NC4A2 strain is referred to as wild-type in this paper. Both the parental wild-type and the *cpnA*- cells were transformed with the pTX-GFP plasmid by electroporation [37]. In addition, both the parental wild-type and *cpnA*- cells were transformed by electroporation with three different plasmids carrying lacZ constructs (*ecmAO*-Gal, *ecmB*-Gal, *pspA*-Gal) obtained from the Dicty Stock Center [24-26].

*Dictyostelium *cells were cultured on Petri dishes in HL-5 media (0.75% proteose peptone, 0.75% thiotone E peptone, 0.5% Oxoid yeast extract, 1% glucose, 2.5 mM Na_2_HPO_4_, and 8.8 mM KH_2_PO_4_, pH 6.5) with penicillin-streptomycin at 20°C. *cpnA*- cells were also cultured in HL-5 media supplemented with 30 μg/mL blasticidin and all plasmid transformed cells were cultured in HL-5 media supplemented with 50 μg/mL G418.

### Dictyostelium Development

*Dictyostelium *cells growing on Petri dishes in HL-5 at 20°C were washed three times in Development Buffer (5 mM NaHPO_4_, 5 mM KH_2_PO_4_, 1 mM CaCl_2_, 2 mM MgCl_2_, pH 6.5). Cells were resuspended in Development Buffer (DB) at 5 × 10^7 ^cells/mL and plated on either black or white filters (Millipore; catalog no. HABP04700 and HAWP04700) placed on pads soaked in DB in dishes (Fisher; catalog no. 09-753-53C). Cells on filters were incubated at 20°C in a humid chamber in the dark for 24 to 48 hours. For cells developed in EGTA, the DB was made without CaCl_2 _and MgCl_2_, and 2 mM or 5 mM EGTA was added. For the control experiment, DB was made with 2 mM EGTA and 4 mM CaCl_2_. For the mixing experiments, wild-type and *cpnA*- cells were mixed at various ratios before plating on black filters.

### X-Gal Staining of Developing Structures

*Dictyostelium *cells were developed on white filters as described above. At various time points in development, 1/6 of the filter was cut and removed for fixing and staining. Filter pieces were placed in Petri dishes in a fume hood and developing structures were fixed with 1% glutaraldehyde and 1 mM EGTA in Z-buffer (60 mM Na_2_HPO_4_, 40 mM NaH_2_PO_4_, 10 mM KCl, 1 mM MgSO_4_) applied with an aerosol spray bottle. After 10 minutes, filter pieces were moved to a 6-well plate and washed once with Z-buffer using a micropipette. To permeabilize the cells, a 1% NP-40 solution was transferred onto the filters using a pipette and allowed to incubate for 10 minutes. The filter pieces were washed with Z-buffer to remove the NP-40 solution and the filters were allowed to dry for 20 minutes. Next, the staining solution (5 mM K_3_Fe(CN)_6_, 5 mM K_4_Fe(CN)_6_, 1 mM EGTA, 2 mM X-Gal in Z-buffer) was pipetted into the well containing the filter and the plate was placed in the dark for 15-30 minutes. The filter pieces were then washed once with Z-buffer and incubated with stop stain solution (50% methanol/50% Z-buffer) for 10 minutes. Next, a 0.02% Eosin Y solution was added to act as a counter stain for 5-10 minutes. The excess counter stain was removed and the filters were placed again in the stop stain solution.

### Microscopy

Images of developing *Dictyostelium *on black filters were taken using a Leica fluorescence dissecting microscope (model no. MZ16F) at 20×, 40×, or 50× magnification using brightfield or fluorescence microscopy and a Leica DFC340FZ camera with the Leica Application software. For time-lapse imaging, cells expressing GFP were plated on black filters on pads in a 60 mm dish. The 60 mm dish was placed in a 100 mm dish with water to keep the plate from drying out. Fluorescence images using the filter for GFP were taken every 10 minutes at 20× magnification over a 28-hour period. Images were converted to movie format using the Lecia Application software. Images of X-Gal stained developing *Dictyostelium *structures were taken with a Nikon SMZ1500 dissecting microscope equipped with a SPOT Flex colored camera using the SPOT digital imaging software. For the imaging of stalk and spore cells, fruiting bodies or stalk structures were removed from the filters using tweezers and placed on glass bottom dishes containing DB buffer. The stalk and spores cells were imaged with differential interference contrast (DIC) and wide field fluorescence on a Nikon TE2000 with a 60× or 100× objective, a Cooke Sensicam camera, a filter cube for GFP, and Image Pro-Plus software.

## Abbreviations

CpnA: Copine A; GFP: Green Fluorescent Protein; EGTA: Ethyleneglycol-O, O'-bis(2-aminoethyl)-N, N, N', N':tetraacetic acid; DIC: differential interference contrast; X: Gal- -bromo-4-chloro-3-indolyl-b-D-galactopyranoside; EcmB: Extracellular matrix protein B; EcmA: Extracellular matrix protein A; PspA: prespore specific protein A; lacZ: β-galactosidase.

## Authors' contributions

TSS carried out all of the experiments and captured the images except for the lacZ construct experiments in Figure 3. JMP and ACD carried out the LacZ experiments and captured the images in Figure 3. TSS, JMP, and ACD contributed to the writing of the figure legends and methods section and assisted in creating the figures. CKD designed and supervised the experiments, created the figures, and wrote the manuscript. All authors have read and approved the manuscript.

## Supplementary Material

Additional file 1 **Time-lapse imaging of developing wild-type *Dictyostelium *cells**. Wild-type *Dictyostelium *cells expressing GFP were plated on black filters in starvation buffer at 5 × 10^7 ^cells/mL and imaged every 10 minutes for 28 hours using a Leica dissecting fluorescence microscope at 20× magnification. Individual images were converted to an .avi movie file at 2 frames/sec, then converted to a mpeg file. Click here for file

Additional file 2 **Time-lapse imaging of developing *cpnA- Dictyostelium *cells**. *cpnA- Dictyostelium *cells expressing GFP were plated on black filters in starvation buffer at 5 × 10^7 ^cells/mL and imaged every 10 minutes for 28 hours using a Leica dissecting fluorescence microscope at 20× magnification. Individual images were converted to an .avi movie file at 2 frames/sec, then converted to a mpeg file. Click here for file
